# Randomised controlled trials and clinical maternity care: moving on from intention-to-treat and other simplistic analyses of efficacy

**DOI:** 10.1186/1471-2393-13-15

**Published:** 2013-01-17

**Authors:** Welsh AW

**Affiliations:** 1 Division of Obstetrics and Gynaecology, School of Women’s & Children’s Health, University of New South Wales, Randwick, NSW, 2031, Australia; 2 Department of Maternal-Fetal Medicine, Royal Hospital for Women, Barker Street, Randwick, NSW, 2031, Australia; 3 Australian Centre for Perinatal Science (ACPS), University of New South Wales, Randwick, NSW, 2031, Australia

**Keywords:** RCT, ITT, Method-effectiveness, Clinical efficacy, Clinical effectiveness, Instrumental variable

## Abstract

**Background:**

The obstetrical literature is dominated by Randomised Controlled Trials (RCTs), with the vast majority being analysed using an intention-to-treat (ITT) approach. Whilst this approach may reflect well the consequence of assignment to therapy and hence the ‘trialists’perspective’, it may fail to address the consequence of actually receiving therapy (the patient’s perspective).

**Discussion:**

This review questions the ubiquitous adherence to the ITT approach, and gives examples of where this may have misled the maternity care professions. It gives an overview of techniques to overcome potential deficiencies in result presentation, using method effectiveness models such as ‘Per Protocol’ (PP) or ‘As-Treated’ (AT) that may give more accurate clinical meaning to the presentation of obstetrical results. It then proceeds to cover the added benefits, considerations and potential pitfalls of the use of Instrumental Variable (IV) models in order to better reflect the clinical context.

**Summary:**

While ITT may achieve statistical purity, it frequently fails to address the true clinical or patient’s perspective. Though more complex and potentially beset by problems of their own, alternative methods of result presentation may better serve the latter aim. Each of the other methods may rely on untestable assumptions and therefore it is wisest that study results are presented in multiple formats to allow for informed reader evaluation.

## Background

### Intention-to-treat (ITT) analysis

In the hierarchy of statistical trials, the RCT has been on the throne for over half a century [1]. Bradford-Hill is credited with provision of ‘criteria’ inferring causation [2], that have guided RCT design since 1965, though it is unclear whether this was his intention [3,4]. The universally favoured method for data analysis from RCTs is ITT [5,6], proposed for two reasons: ensuring comparable treatment groups due to randomisation, and preventing bias in analyses resulting from post randomisation exclusions. With ITT a solid statistical basis is said to exist for determining the significance of any observed difference in outcome between treatment groups [7], though all patients must be followed according to a prespecified schedule of outcome measurements regardless of compliance, adverse effects or other post randomisation observations [8].

Randomisation and adherence/compliance are critical for ITT to produce a good estimate of efficacy. Randomisation “provides a guarantee against biased estimates of treatment effects due to differences in the distribution of known and unknown confounding factors” [7]. ITT produces unbiased estimates of the effects of receiving a treatment only when subjects are willing to adhere to all treatments under study and complete all planned outcome assessments [9]. Data that are missing from randomized patients must not bias the comparison of treatment groups and outcome measurements must be obtained in a like and unbiased manner for all patients [8].

## Discussion

### Pitfalls of ITT

ITT can successfully provide valid estimates for the effect on outcome of assignment to therapy, though these reflect the effect of randomisation to treatment rather than the true effect of taking treatment [10]. Though this works well in an ‘ideal’ statistical situation, problems arise when ITT is applied to complex clinical scenarios. The expression ‘treatment contamination’ may be used for the common situation of RCT violations whether through non-compliance (not receiving the treatment or intervention) or crossover (switching to receive that of the other group) [11]. Traditional implementation of ITT assumes all subjects to be latent compliers (possessing a disposition to comply) [9], which rarely occurs in clinical practice particularly when subjects may have strong views about which treatment they want. When this can be ascertained before randomisation, these subjects should not be included in a trial [7]. This is highly pertinent for maternity care where patients are likely to be highly informed. The significant non-compliance seen in maternity studies cannot be assumed to be random and so introduces profound selection bias where patients chose certain treatments or protocols [12]. Thus the perspective of society may be that ITT analysis is appropriate for RCTs, but this may not be appropriate from the patient’s perspective.

In clinical practice there are frequent deviations from ‘ideal’ trial conditions, provoking compromises in RCT design to try and overcome this disconnect. Pragmatic trials compare two treatment strategies and tend to reflect the situation in current clinical practice, using less selected participants and being conducted under more realistic conditions with lower adherence to the assigned treatment [13]. Explanatory trials concentrate on compliant patients in order to study the biological effect of the treatment. Many studies end up as a compromise between pragmatic and explanatory, and large trials of long duration often start as exploratory questions and end up as a comparison of two strategies. Many study designs, like large simple trials, are better described as longitudinal studies with baseline randomisation, rather than as either pure randomised or observational studies. As soon as there are deviations from protocol in an RCT, then investigators are able only to record data as if they were conducting a prospective observational study – the more deviation, the more like an observational study it becomes [14]. This is more commonly seen with studies with long follow-up periods. Often a ‘pseudo-ITT’ analysis is performed restricted to subjects with complete data or techniques are employed such as ‘last available observation carried forward’ or ‘complete-case’ evaluations, which are not true ITT [14]. Both of these weaker versions of ITT assume no selection bias due to incomplete follow up.

A further alternative, when the issue is that of missing data resulting in incomplete adherence to ITT, is the use of (multiple) imputation analysis (MIA), a technique used to generate plausible values for missing data, using a set of rules for combining individual estimates and standard errors [15]. Guidelines for combining estimates of interest have previously been outlined [16], though previous studies have shown that despite its increasing use, it is rarely adequately reported and potentially inappropriately applied [17]. Pitfalls of MIA include omitting outcome variables after imputation; non-normally distributed variables; the assumption that missing data are random; computational and practical limitations [18].

### Efficacy and effectiveness

Studies may aim to determine *clinical efficacy* (“how well a treatment works under perfect adherence and highly controlled conditions” [13] or the patient’s perspective on a trial) or *clinical effectiveness* (“how well a treatment works in everyday practice” [13], or society's perspective on a trial” [13]). For clinical practice, an assessment of effectiveness that accounts for patient compliance must be considered alongside that of efficacy [10], given the knowledge that a certain percentage will not comply with treatment in both the clinical and research settings. Given that some of the most critically important clinical questions are addressed using RCTs with ITT, where there are always issues of ‘study purity’ and noncompliance, one may wonder why ITT remains so dogmatically adhered to. A number of reasons have been proposed including: statistical simplicity; belief that all analyses not based on original assignment are invalid; preference for simple analyses with easily understood conclusions compared to the subjective complexity of model based analyses; model-based analyses often involve considerable judgement and relatively complex and tedious calculations, requiring appropriate skill, computation and software [10].

ITT does not reflect the whole complexity of patient care and clinical events and may not appear satisfactory unless it yields a positive result. The comparison using ITT is regarded by most as conservative, with most commonly a bias towards the null hypothesis, or dilution due to contamination of the treatment groups [10,13]. The ITT ‘ideal’ for RCT data analysis falls far short of the ideal of true clinical effectiveness, which is what the person considering treatment actually needs to know. As stated in one statistical editorial: “Life would be that simple were it not for human beings” [19].

### ITT in the obstetrical literature

In the trade-off between bias and precision, different schools of thought exist and to date ITT has been almost ubiquitous in the obstetrical literature, despite its apparent shortfalls. One of the clearest and most clinically detrimental examples of this is the Term Breech Trial, where ITT contributed to conclusions that do not appear to be clinically justified, were unsubstantiated at 2 year maternal and neonatal follow up and have had worldwide ramifications in maternity care [20-22]. The criticisms of this study and its deviations from ITT are too extensive to list here but have been detailed well in previous publications [23,24]*.* Also, literature regarding use of epidurals for analgesia in labour has been clouded by a blind reliance on ITT in RCTs, even in the face of significant crossover. In a major RCT comparing epidural with non-epidural analgesia, 31% of women counselled agreed to participate, with 42% of those agreeing in the antenatal clinic being randomised, then 33% not receiving their intended epidural and 28% from the non-epidural group receiving an epidural [25]. The principles of randomisation and adherence were not preserved in this study, yet blind belief in the power of ITT led to the conclusion that “Despite a significant proportion of women in each group not receiving their allocated analgesia, a significant difference in terms of instrumental delivery rates remained” [25]. When multiple RCTs with strict (and often flawed) ITT analysis become combined into Cochrane reviews, which are then held up to be the ‘highest possible form of evidence’ it is no wonder that evidence-based medicine as currently presented [26] has both fans and critics [27]. ‘Clinical trialists’ still hold ITT as the statistically and therefore academically correct method for evaluation of data in RCTs [28], though it is clear that adherence to ‘ideal’ methodology guidelines is suboptimal even for high-impact journals [29].

### Alternatives to ITT: method-effectiveness (ME) models

When evidence-based medicine needs to be applied to real clinical cases and to the patient’s perspective, ME models have been proposed to be more relevant to clinical decision making than efficacy studies [10]. They may more accurately reflect “*human beings and the real world”*, and allow a number of behavioural evaluations [7]. Common simple ME models include *Per Protocol* (PP) or *As-Treated* (AT) approaches. Both make the assumption that the probability of taking the treatment is random with respect to all predictors of outcome [30]. Clinical trialists are suspicious about departures from ITT because the typically hard issues of observational studies then surface in randomized trials [19], it takes further care and skill to move beyond ITT and entrenched approaches are frequently hard to shift.

A PP analysis includes all subjects who were, in retrospect, eligible for enrolment in the study without major protocol violations, who received an acceptable amount of test treatment, and who had some minimal amount of follow up [31]. Selection bias comes in if the reasons that influence participants in compliance with their assigned treatment are associated with prognostic factors [13]. It has been recently estimated that the PP estimate (log odds ratio (OR)) is 1.25 times the ITT estimate [32].

The ultimate goal of outcome research is to tell which treatment or intervention is associated with the best outcome, whether maximum benefit or minimum harm to patients. As this may involve establishing if there is a causal association between that treatment or intervention and outcome, researchers concerned with the effects of non-compliance have often used the AT approach [9], which analyses subjects according to treatment received, not assigned [31]. It attempts to deal with aspects of the power calculation issue of PP analysis by using all the data, at the price of blurring the definition of adherence to treatment, but it does nothing about the causality confusion created by the analysis-stage redefinition of treatment groups that may create prognostically distinct populations. If one is truly after a valid answer to the question of ‘method-effectiveness’ then it has been suggested by some that ITT and PP will not supply a valid answer, and only AT may do so, though at a cost [10]. Concerns regarding AT approaches include: lost randomisation; potential for biased results; inability to identify and account for important prognostic factors; impossibility of generalisation [33].

Unless alternative methods are considered through the RCT process, blind adherence to ITT risks becoming a self-fulfilling prophecy [10]. The data that would help estimate method effectiveness are not gathered, and one has little recourse but to settle for estimates of use-effectiveness, which might, as already noted, be of little value even for projecting use-effectiveness in the future. Unfortunately, whilst simple PP and AT analyses may appear to reflect the patient’s perspective more clearly, these techniques also leave a bit to be desired. Whereas ITT most commonly suffers from ‘bias towards the null’ [12,16,17], there are unpredictable confounding biases in AT analysis and selection biases in PP analysis that can go in either direction depending upon multiple factors. The estimates from these analyses can only be interpreted as the effect of treatment if the analysis is appropriately adjusted for identifiable confounders [13]. This therefore means that one must adopt some more or less elaborate “model” as simple data summary will not suffice. Models (which require untestable assumptions) mean increased subjectivity and uncertainty though in contrast the price for insisting on greater objectivity and certainty (using only ITT) is that one is less clinically relevant [10]. The clinician may be caught between interpretations of effectiveness versus efficacy, wishing to know the effect on their patient if they actually follow a particular treatment (efficacy), while recognising that potential non-compliance means that measures of effectiveness may have more meaning. It may be appropriate to include in clinical counselling the fact that a given percentage will not comply with a particular treatment course (influencing the ITT result) but that if the patient does persist that a particular effect is to be anticipated (the PP result).

### Causal inference approaches and instrumental variable models

There are a number of different causal inference approaches that adjust for unmeasured confounders (i.e. hidden bias) in order to improve upon the shortcomings of the method-effectiveness models. These include inverse probability (IP) weighting, g-estimation and instrumental variable (IV) methods [14]. Inverse probability (IP) weighting or g-estimation are generally used for confounding adjustment in AT and PP analyses involving time-varying treatments and require untestable assumptions similar to those made for causal inference from observational studies [13]. If only sequential randomisation of treatment within levels of measured covariates is assumed, then IP weighting is needed. If only a dose–response model is assumed then g-estimation is needed [14]. If both of these assumptions are made then either technique may be used.

The *instrumental variable* (IV) model, a particular form of g-estimation that does not require measurement of any confounders is a commonly used causal approach to estimate the true trial effect [13], and may be used to obtain for each patient a predicted probability of receiving the experimental treatment [34]. It is often regarded as a more useful approach than ITT if method effectiveness is the primary study goal [10]. The IV approach requires the use of at least 1 ‘instrument’ which is a variable that is powerful and valid and correlates with treatment but is uncorrelated with unobserved determinants of the dimension of health or clinical endpoint under study [35]. It does not require measurement of confounders, and makes the exposure of interest more or less likely (in a similar manner to randomisation) but does not affect the outcome [36]. The IVs are assumed to mimic randomisation variables and for each patient they obtain a predicted probability of receiving the experimental variable [34]. It must be stressed, however, that IV models rely on untestable assumptions, so while they may have increased clinical validity, they need to be treated with caution, and it is recommended that they are not the only analysis done in an RCT. Examples of the instrument include the randomisation method of an RCT, the cost of a treatment or regional variations in treatment availability. In Mendelian randomisation, genotypes may act as IVs [37]. An IV adjusted ITT treats the RCT as an IV with treatment assignment being the instrument, and the effect of this assignment on outcome is adjusted by the percentage of assigned participants who receive the treatment [11].

Whist technically more complex, a growing body of literature supports and explains the use of IV methods to determine effectiveness in a number of trial situations [12,36], correcting for non-compliance based on assumptions about outcomes for non-compliers under both treatments [38]. A recent publication has tabulated the strengths and weaknesses of the different methods of RCT analysis, proposing use of IV to adjust for treatment contamination [11]. These different approaches have been presented in many fields, such as an extensive comparison of the ITT, AT and IV approaches in psychiatry [39], though have failed to penetrate the obstetrical literature. Further details of the mathematical and statistical techniques for ME assessment as applied to longitudinal studies (g-estimation, IP and IV) have been outlined in a recent review [14]. This review clearly tabulates the influence of the different forms of analysis on effect estimates and 95% confidence interval in a study on atypical antipsychotic medication. A more mathematical discussion of IV methods and RCTs has been presented by Dunn et al. [40].

As well as the IV approach, researchers have proposed the use of four mutually exclusive groups using a new estimator referred to as a stratified method of moments estimator [7,9]. These are: compliers; always-take-experimental treatment; never-take-experimental treatment; defiers (always take the opposite). Estimating proportions and likelihoods for each of these groups may make assumptions that hold better in randomised trials and less so in the observational setting. Methods to estimate and overcome bias are difficult to perform and may all need assumptions to be made that cannot be directly measured. It is clear that there is no single perfect method for presentation of data from all RCTs. All model assumptions need to be articulated explicitly, their validity tested against the particulars of each trial.

## Summary

Whilst RCTs provide the most scientifically and statistically accurate method for evaluating the influence of treatment or interventions, obstetrical studies are dominated by the use of ITT analysis to present their data. In fact, though ITT may correlate well with the scientific or statistical aims of a study or of society, it frequently does not answer the needs of the patient. Deviations from pure adherence to the intervention under evaluation undermine the value of ITT, and mean that patients may benefit from alternative methods of data presentation.

In publishing RCT results, supplementation of ITT evaluation with ‘As Treated’ or ‘Per Protocol’ starts to provide more relevant information for patients [41], especially when there is substantial lack of adherence or loss to follow-up, though AT and PP are relatively crude tools with their own deficiencies. Further clinical relevance may come from use of Instrumental Variable methods though techniques such as IV rely upon the use of untestable assumptions, so should be treated cautiously and not used in isolation.

These concepts have progressed well in many disciplines but are yet to be reflected in obstetrical medicine and maternity care, where non-compliance and treatment contamination are so common. A number of well publicised trials have misled clinical practice because of over-reliance on ITT in the face of non-adherence after randomisation. It therefore would be more appropriate to present results of randomised controlled trials in multiple formats to allow clinicians to discuss both the statistical and clinical relevance of findings. It is clear that there is no single perfect method for presentation of data from RCTs, and each should be approached with clear knowledge of their limitations.

## Abbreviations

RCT: Randomised controlled trial; ITT: Intention to treat; ME: Method-effectiveness; PP: Per protocol; AT: As-treated; IV: Instrumental variable; IP: Inverse probability; MIA: Multiple imputation analysis.

## Competing interests

The author declares that they have no competing interests.

## Author’s contributions

AW devised and wrote the paper.

## Pre-publication history

The pre-publication history for this paper can be accessed here:

http://www.biomedcentral.com/1471-2393/13/15/prepub
